# Epidemiology and outcomes of carbapenem-resistant *Klebsiella pneumoniae* infections in patients with hematological malignancies from 2014 to 2022

**DOI:** 10.3389/fmicb.2024.1507908

**Published:** 2025-01-07

**Authors:** Meng Li, Liyan Ye, Zhenghao Yu, Hongwu Yao, Yunxi Liu, Guanglei Wang, Mingmei Du

**Affiliations:** ^1^Department of Hematology, The First Medical Center of Chinese PLA General Hospital, Beijing, China; ^2^Department of Laboratory Medicine, The First Medicine Center of Chinese PLA General Hospital, Beijing, China; ^3^Health Management Institute, The Second Medical Center and National Clinical Research Center for Geriatric Diseases, Chinese PLA General Hospital, Beijing, China; ^4^Department of Infection Management and Disease Control, The First Medical Center of Chinese PLA General Hospital, Beijing, China; ^5^Ministry of Guards, The First Medical Center of Chinese PLA General Hospital, Beijing, China

**Keywords:** epidemiology, *Klebsiella pneumoniae*, carbapenem-resistant, hematological malignancies, infections

## Abstract

**Background:**

We aimed to describe the epidemiology, cross-transmission, interventions, and outcomes of carbapenem-resistant *Klebsiella pneumoniae* (CRKP) infections in the hematological malignancies (HM) department of a hospital in China.

**Methods:**

This prospective study was divided into three stages from 2014 to 2022: Period 1 (from 1 January 2014 to 4 March 2021), Period 2 (from 5 March 2021 to 31 December 2021), and Period 3 (from 1 January 2022 to 31 December 2022), with different measures implemented at each stage to evaluate the rate of new infections. The risk factors, epidemiological characteristics, data from all patients with CRKP, NGS gene sequencing molecular epidemiology analysis, and cross-transmission were described.

**Results:**

A total of 217 patients with *K. pneumoniae* isolates, including 38 (17.5%) patients with CRKP isolates, were confirmed in the HM department. The total rate of CRKP in KP isolates was 17.5%. The predominant clone in the HM department was ST11 CRKP producing the KPC-2 enzyme (21, 70.0%). A total of 23 cases of HM department-acquired CRKP infections were identified, and five hospital cross-transmission events were observed. Four instances of CRKP dissemination were primarily related to clone ST11. Only one outbreak occurred at the end of Period 1, involving four cases of CRKP healthcare-associated infections (HAIs). After the implementation of outbreak intervention bundles at the end of Period 2, no cross-transmission occurred. The rate of CRKP in KP isolates decreased to 12.9% (only four CRKP isolates) in Period 3, down from a peak of 46.7% in Period 2 (including 14 CRKP isolates). Only one new carrier (0.9%) was identified during the two cross-sectional active screenings of the rectal swab. The 28-day mortality rate was 38.7% (12/31) in CRKP-infected patients.

**Conclusion:**

The prevalence of CRKP in the HM department was relatively low in the studied hospital. We found that interventions, including single-room isolation, enhanced disinfection, and skin decolonization, played a pivotal role in controlling the spread of HM-acquired CRKP infections.

## Background

Global carbapenem-resistant *Klebsiella pneumoniae* (CRKP) infections have shown progressive and exponential growth in recent years. Patients with hematological malignancies (HM) or hematopoietic stem cell transplant (HSCT) recipients may be particularly vulnerable to CRKP infections because of chemotherapy-induced gastrointestinal mucositis, prolonged hospitalizations, neutropenia, and the frequent use of broad-spectrum antibacterial agents (Pouch and Satlin, 2017). Infections caused by CRKP are associated with high morbidity and mortality rates, particularly in HM or HSCT patients.

A previous study revealed that patient-to-patient transmission of CRKP accounts for an estimated 52% of the cases identified in healthcare settings (Harris et al., 2007). Currently, most studies on CRKP cross-transmission, prevention, and control are generally focused on high-risk departments such as the respiratory department and ICU (Weist et al., 2002; Chi et al., 2022; Li et al., 2019). The effective measures included comprehensive interventions, such as de-escalation and targeted bundle interventions, clarifying the role of separate, small wards within the ICU in controlling the transmission of CRKP (Chi et al., 2022; Li et al., 2019). However, limited information has been reported regarding the epidemiology, cross-transmission, and prevention measures of CRKP infections in the HM department, which could improve the control of CRKP spread among HM patients.

Our previous research showed that the rate of CRKP among the KP isolates was 39.0% in our hospital from 2010 to 2019, while this rate in the ICUs was 65.6% (Li et al., 2022; Li et al., 2023). However, the rate of CRKP among the *K. pneumoniae* isolates in the HM department was 15.0%, which was significantly lower than that in other departments. In this study, we aimed to evaluate the epidemiological characteristics of CRKP, cross-transmission between patients, and the effectiveness of interventions to prevent the spread of CRKP in the HM department.

## Methods

### Setting

The hospital is a tertiary teaching hospital with 3,800 beds, located in Beijing, northern China. There are 125 beds in the HM department, distributed among one HSCT ward (including 20 beds) and three other wards (35 beds in each ward). Ward 1 mainly accommodates leukemia patients, Ward 2 mainly accommodates non-leukemia patients (e.g., lymphoma or multiple myeloma), Ward 3 mainly accepts patients after transplantation, and Ward 4 is the HSCT ward. There are approximately 120 transplant patients in the HSCT ward and 2,000 patients receiving treatment in the HM department every year.

### Definitions

Patients with positive CRKP detection, in the absence of any signs or symptoms of infection, were defined as colonized with CRKP. Healthcare-associated infection (HAI) was defined as an infection occurring more than 48 h after patient admission to the hospital. HM department-acquired CRKP infection was defined as the first positive culture identified more than 48 h after admission to the HM department. A CRKP outbreak was defined as the occurrence of more than three CRKP HAI cases in the HM department within 2 weeks. The 28-day mortality was defined as death from any cause within 28 days of *K. pneumoniae* infection.

### Data collection

From January 2014 to December 2022, patients who were admitted to the HM department were enrolled if they had one episode of CRKP. Only the first CRKP detection for each patient was included, and recurrent detections were excluded. The collected data included age, sex, diagnosis on admission, hospital stay before CRKP detection, length of hospitalization, antimicrobial drug exposure (referring to the use of antibiotics within 30 days before *K. pneumoniae* detection), prior stay in an ICU or other department, prior chemotherapy or radiotherapy within 30 days, previous HSCT, and outcomes. A case–control design was used to analyze risk factors for CRKP in patients with *K. pneumoniae* in the HM department, and descriptive analysis was employed to study the epidemiological characteristics of CRKP.

### Prevention and control interventions

The study was conducted in three stages.

1. Period 1 (from 1st January 2014 to 4th Mar 2021): This was a 7-year baseline period during which simple interventions were implemented. These included contact precautions, bedside isolation of the patients with CRKP, personnel training, hand hygiene, environmental disinfection, and the monitoring and reporting of HAIs.
2. Period 2 (from 5th March 2021 to 31st December 2021): In addition to the interventions from Period 1, this period also included outbreak control intervention bundles, such as single-room isolation of the patients with CRKP (isolation gown, gloves, shoe covers); enhanced medical staff education; isolation of the patients transferred from the ICU or other hospitals with a history of infection in a single room until negative cultures were obtained; terminal disinfection with hydrogen peroxide once a month in each room or for the patients with CRKP after discharge; and active screening of the rectal swabs during two cross-sectional periods in March and July 2021.
3. Period 3 (from January 1st 2022 to December 31th 2022): In addition to the interventions from Period 2, the hospital implemented special prevention and control measures for CRKP activity in all departments. The patients transferred from the ICU were required to first take a bath or scrub themselves with chlorhexidine gluconate. Additional education was provided to the cleaning staff and patient companions, and adenosine triphosphate (ATP) bioluminescence testing and the patients’ surrounding sampling were conducted to assess the quality of environmental cleaning. The implementation of the CRKP prevention and control measures in each department was supervised, evaluated, and reported on once a month by infection control practitioners (ICPs).

### Microbiological studies

The *K. pneumoniae* isolates were cultured using the BacT/ALERT® 3D™ system (bioMérieux SA, Marcy-l’Etoile, France) or on blood agar culture plates in the microbiology laboratory. All isolated *K. pneumoniae* were identified using VITEK MS (bioMérieux SA, Marcy-l’Etoile, France) and FastANI v1.32, with a 95% average nucleotide identity (ANI) threshold. Lists of antimicrobial categories proposed for antimicrobial susceptibility testing were created using documents and breakpoints from the annually updated Clinical Laboratory Standards Institute (CLSI) 2023-M100-S33 edition. Carbapenem resistance was defined as an MIC of ≥2 μg/mL for ertapenem or MIC ≥4 μg/mL for meropenem or imipenem. The isolates that exhibited non-susceptibility to carbapenems were screened by PCR amplification for the common carbapenemase genes, namely, *bla*_KPC_, *bla*_NDM_, *bla*_OXA−48_, and *bla*_IMP,_ as described previously (Poirel et al., 2011).

### Whole genome sequencing (WGS) and genomic data analysis

Whole genome sequencing (WGS) was performed with a paired-end library, with an average insert size of 350 bp, on the HiSeq X Ten sequencer (Illumina, California, CA, United States). The draft genome was assembled into a scaffold. Quality assessment was performed with Fastqc (Version 0.11.8), and all reads with a score above Q30 were used for follow-up analysis. After removing the adapter and barcode and trimming the raw reads, the sequences were assembled using SOAPdenovo (SOAP version 2.21) with default settings. Resistance genes, plasmid replicon types, and multi-locus sequence types (STs) were predicted using ResFinder v2.1, PlasmidFinder v1.2, and mlst v2.0, respectively (for *K. variicola*, the *K. variicola* platform was used^1^). The virulence genes were analyzed using the Kleborate platform. The phylogenic tree based on single-nucleotide polymorphisms (SNPs) was constructed using CSI Phylogeny 1.4^2^ with default settings and was visualized and annotated using iTOL. The complete genome sequence of the *K. pneumoniae* strain HS11286 (GCF_000240185.1) was used as a reference.

### Ethics

This study was reviewed and approved by the Medical Ethics Committee of PLA General Hospital (Reference number: S2019-142-02), which was conducted in accordance with the Declaration of Helsinki.

### Statistical analysis

To evaluate categorical variables, the *χ*^2^ test or two-tailed Fisher’s exact test was used as appropriate. Continuous variables were analyzed using Student’s *t*-test (for normally distributed variables) or the Mann–Whitney U test (for variables that were not normally distributed). For the continuous variables, the results were expressed as median (interquartile range [IQR]) or mean ± standard deviation (SD), and the categorical variables were expressed as percentages. All variables with a *p*-value of <0.05 in the univariable analysis were included in a multivariable backward logistic regression analysis. The annual change trend in the rate of CRKP among the *K. pneumoniae* isolates from 2014 to 2022 was analyzed using *p* for trend. A *p*-value of <0.05 was considered statistically significant. Statistical analysis was performed using IBM SPSS version 22.0 (IBM Corp., Armonk, NY, USA).

## Results

### Patient characteristics

A total of 217 patients with *K. pneumoniae* isolates, including 38 (17.5%) patients with CRKP isolates, were confirmed in the HM department. The median age of the patients was 48 years, and 65% of the participants were male. A total of 154 (71%) episodes were CRKP HAIs, and 44 (20.3%) were HM department-acquired CRKP infections. Considering the isolated samples, 98 (45.2%) episodes were from the primary respiratory tract, followed by BSIs (30.9%) and urinary tract infections (13.8%).

We compared the clinical characteristics of the patients with CRKP and carbapenem-sensitive *K. pneumoniae* (CSKP) infections (Table 1). The factors found to be significantly associated with CRKP, based on the univariate analysis, included the HSCT recipients, prior ICU stay, use of a central venous catheter, and carbapenems. In the multivariate analysis, the HSCT recipients (OR = 0.36, 95%CI: 0.13–0.96), carbapenems (OR = 2.89, 95%CI: 1.22–6.83), and prior ICU stay (OR = 26.78, 95%CI: 3.08–233.09) were identified as independent factors for CRKP development (Table 2).

**Table 1:** Baseline characteristics of the patients with CRKP and CSKP.

Characteristics	Overall (*n* = 217)	CRKP (*n* = 38)	CSKP (*n* = 179)	*p*-value
Age, median (IQR), year	48 (30–59)	48 (28–60)	48 (31–58)	0.806
Sex, males, *n* (%)	141 (65.0)	20 (52.6)	121 (67.6)	0.117
Sample type detected by KP, *n* (%)				
Blood	67 (30.9)	17 (44.7)	50 (27.9)	0.061
Respiratory tract	98 (45.2)	12 (31.6)	86 (48.0)	
Urinary	30 (13.8)	3 (7.9)	27 (15.1)	
Others				
Colonization or infection, *n* (%)				
Colonization	59 (27.2)	7 (18.4)	52 (29.1)	0.181
Infection	158 (72.8)	31 (81.6)	127 (70.9)	
Healthcare-acquired infection, yes, *n* (%)	154 (71.0)	23 (60.5)	131 (73.2)	0.172
HM department-acquired KP, yes, *n* (%)	44 (20.3)	21 (55.3)	23 (12.8)	<0.001
Diagnosis on admission, *n* (%)				
Acute leukemia	127 (58.5)	26 (68.4)	101 (56.4)	0.383
Lymphoma or multiple myeloma	64 (29.5)	9 (23.7)	55 (30.7)	
SAA or MDS	23 (10.6)	2 (5.3)	21 (11.7)	
Others	3 (1.4)	1 (2.6)	2 (1.1)	
HSCT recipients, *n* (%)				
Allogeneic transplant	78 (35.9)	9 (23.7)	69 (38.5)	0.073
Autologous transplant	7 (3.2)	0 (0.0)	7 (3.9)	
No	132 (60.8)	29 (76.3)	103 (57.5)	
Hospital stays prior to KP, days (median, IQR)	12 (2, 22)	10 (1, 25)	12 (3, 20)	0.741
Total hospital stays, days (median, IQR)	31 (20–46)	29 (12–46)	31 (21–47)	0.499
Prior chemotherapy or radiotherapy, yes, *n* (%)	164 (75.6)	30 (78.9)	134 (74.9)	0.745
Prior ICU stay, yes, *n* (%)	9 (4.1)	7 (18.4)	2 (1.1)	<0.001
Ventilator, yes, *n* (%)	6 (2.8)	3 (7.9)	3 (1.7)	0.114
Use of venous catheter, yes, *n* (%)	154 (71.0)	32 (84.2)	122 (68.2)	0.075
Use of urinary catheter, yes, *n* (%)	20 (9.2)	4 (10.5)	16 (8.9)	1
Antibiotics use within 30 days before KP isolation				
Use of carbapenems, yes, *n* (%)	81 (37.3)	24 (63.2)	57 (31.8)	0.001
Use of β-lactam-β-lactamase inhibitor, yes, *n* (%)	102 (47.0)	22 (57.9)	80 (44.7)	0.193
Cephalosporin, yes, *n* (%)	21 (9.7)	3 (7.9)	18 (10.1)	0.915
Combination regimens, yes, *n* (%)	10 (4.6)	2 (5.3)	8 (4.5)	1
28-day mortality, *n* (%)				
Survival	197 (90.8)	26 (68.4)	171 (95.5)	<0.001
Death	20 (9.2)	12 (31.6)	8 (4.5)	

CSKP, carbapenem-sensitive *Klebsiella pneumoniae*; IQR, interquartile range; CRKP, carbapenem-resistant *Klebsiella pneumoniae*; KP, *Klebsiella pneumoniae*; SAA, serious-aplastic-anemia; MDS, myelodysplastic syndrome.

**Table 2:** Univariate and multivariable analysis of the risk factor for CRKP in the patients with KP in the HM department.

Characteristics	Univariate analysis		Multivariable analysis	
	OR (95%CI)	*p*-value	OR (95%CI)	*p*-value
HSCT recipients				
No	Ref.			
Yes	0.42 (0.19–0.94)	0.073	0.36 (0.13–0.96)	0.042
Prior ICU stay				
No	Ref.		Ref.	
Yes	19.98 (3.97–100.69)	<0.001	26.78 (3.08–233.09)	0.003
Venous catheter				
No	Ref.		Ref.	
Yes	2.49 (0.99–6.30)	0.075	2.73 (0.85–8.71)	0.091
Carbapenems				
No	Ref.		Ref.	
Yes	3.67 (1.77–7.62)	0.001	2.89 (1.22–6.83)	0.016

HSCT, hematopoietic stem cell transplant; ICU, intensive care unit; Ref, reference; OR, odds ratio; CI, confident interval; KP, *Klebsiella pneumoniae*; HM, hematological malignancies.

### Whole genome sequencing (WGS) and genomic data analysis

**Figure 1:**
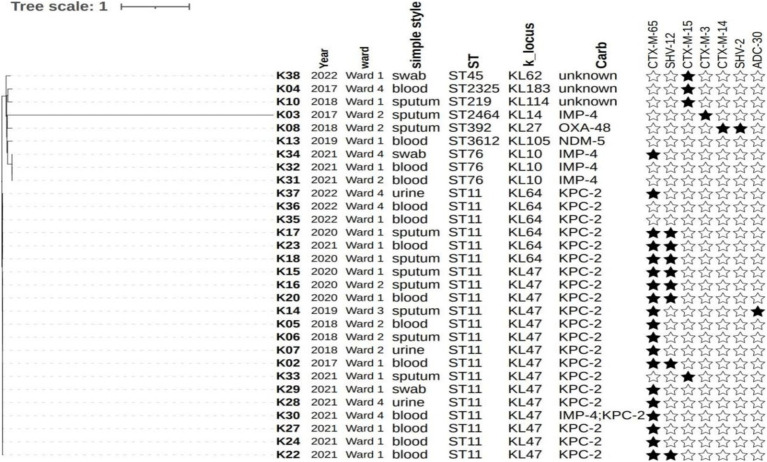
The whole genome sequencing (WGS) and genomic data analysis of CRKP.

WGS of 30 CRKP strains was performed. K3 was identified as *K. variicola* using FastANI. Regarding the classification of the CRKP-producing enzymes, 21 strains were KPC-2, five were IMP-4, one was NDM-5, and one was OXA-48. One isolate co-harbored both bla_IMP-4_ and bla_KPC-2_ and was resistant to all three carbapenems tested. MLST analysis classified the isolates into eight different sequence types (STs), with ST11 being the most prevalent (21/30). Among the ST11 strains, KL47 was the predominant serotype (15/21) and the remaining strains were all KL64. The majority of the ST11 strains coded for yersiniabactin (*ybt 9*, 20/21; ICEKp3, 20/21), aerobactin (*iuc1*, 12/21), and *rmpA2* (12/21), while all were negative in the string test. The three hypermucoviscous strains were all ST76, KL10 type, and all carried the IMP-4 carbapenemase but did not code for any known virulence genes. The predominant clone in the HM department was ST11 CRKP (21, 70.0%). There were seven other types, but only one strain was detected for six of these types. We found that KL47 (28.57%) and KL64 (71.43%) were detected in the ST11 CRKP strains. Phylogenetic analysis showed that all of the ST11 strains and three ST76 strains (K31, K32, and K34) were highly homologous (Figure 1 and Supplementary Table S1). Among the 12 deceased patients, six strains were subjected to WGS, all of which were classified as ST11, KL47.

### The epidemiology and cross-transmission of CRKP in the HM department

**Figure 2:**
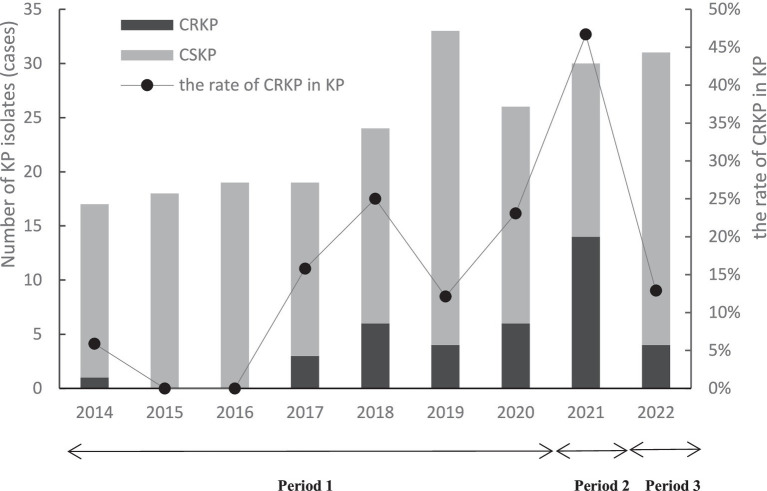
Occurrence of CRKP and CSKP in the HM department during the different intervention periods from 2014 to 2022. Period 1 (from 1st Jan 2014 to 4th Mar 2021): Simple interventions (contact precautions, bedside isolation, personnel training, hand hygiene, environmental disinfection, and monitoring HAIs) were implemented. Period 2 (from 5th Mar 2021 to 31st Dec 2021): In addition to the interventions from Period 1, this phase also included outbreak intervention bundles, such as single-room isolation, enhanced education, isolation of the patients transferred from ICUs or other hospitals in single rooms; terminal disinfection with hydrogen peroxide for the patients with CRKP after discharge; enhanced disinfection using disinfection wipes. Period 3 (from 1st Jan 2022 to 31st Dec 2022): In addition to the interventions from Period 2, the hospital implemented special CRKP control activities in all departments, including the use of chlorhexidine gluconate to scrub the inpatients with CRKP.

The total rate of CRKP in the *K. pneumoniae* isolates was 17.5% (38/217) from 2014 to 2022. The annual rate of CRKP in the *K. pneumoniae* isolates ranged from 5.9% in 2014 to 12.9% in 2022 (Figure 2), which was significant (*p* for trend <0.001). The rate of CRKP in the *K. pneumoniae* isolates peaked in 2021 at 46.7% (including 14 CRKP isolates).

**Figure 3:**
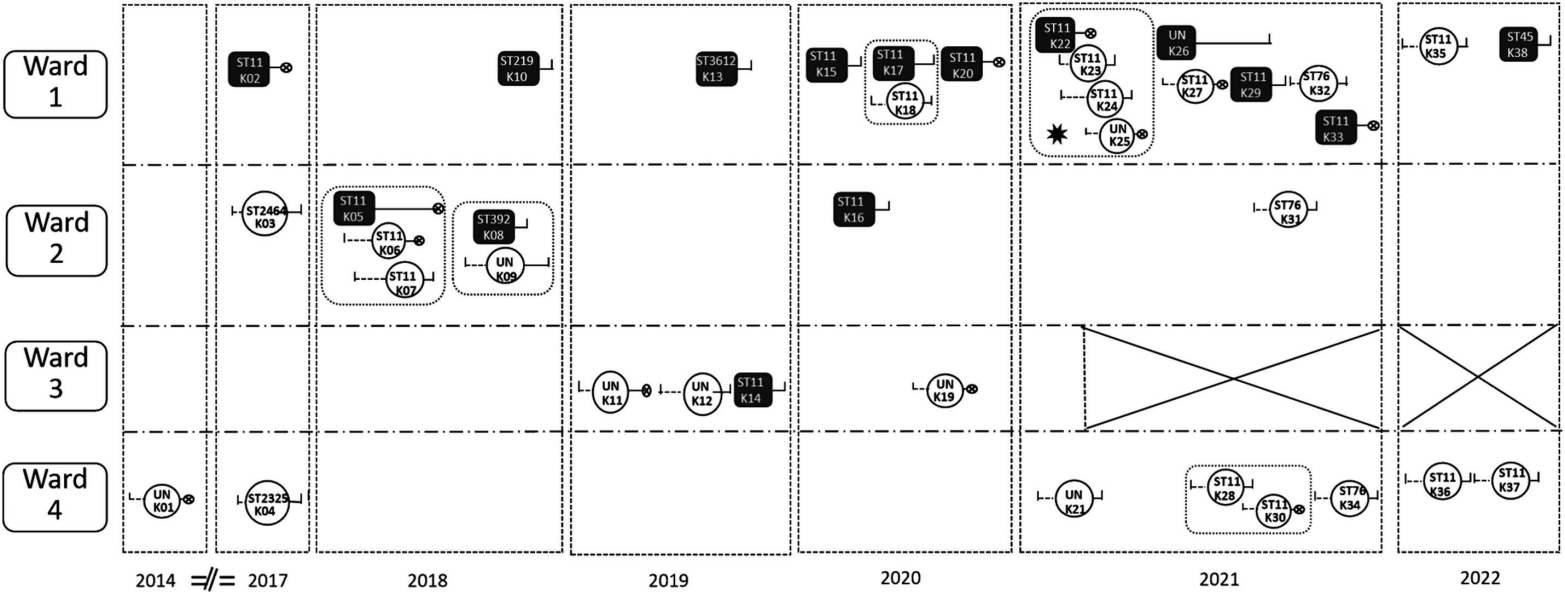
A cross-transmission time–space sequence diagram of the CRKP isolates in the HM department. 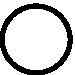: HM department-acquired CRKP infections. 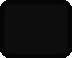: The cases that brought CRKP into the HM department. A total of eight cases were from departments in the studied hospital: K08, K33, and K38 from the Respiratory ICU; K16 and K20 from Respiratory Medicine; K14 and K22 from the Nephrology ICU; and K05 from the comprehensive ICU. All the other six cases were from other hospitals. 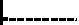: Hospital stay before the CRKP isolation. 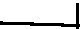: Hospital stay after the CRKP isolation. 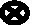: Death of the case. ✸: outbreak. The outbreak occurred from 22nd Feb to 5th March, during the transition period of HM Ward 1and Ward 2. Since then, Ward 3 has been closed. 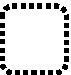:There was an intersection of time in the same ward between case and case. K01, K02, K03……: Chronologically occurring CRKP cases. ST11, ST 2464……: Genotyping of CRKP. UN: These strains were not retained, so no genotyping was performed. Ward 1 mainly accommodates leukemia patients; Ward 2 mainly accommodates non-leukemia patients; Ward 3 mainly accepts patients after transplantation; and Ward 4 is the HSCT ward.

All 38 CRKP cases were HAIs, and 23 cases were HM department-acquired CRKP infections. Of the 15 cases that brought CRKP into the HM department, eight cases were from other departments in the studied hospital and seven cases were from other hospitals. The ward with the most CRKP isolates was Ward 1 (including 18 cases), and the ward with the fewest was Ward 3 (including four cases). A total of five hospital cross-transmission events were observed, and four CRKP dissemination events were primarily related to clone ST11, while only one dissemination event was restricted to clone ST392 between the cases K08 and K09. A cross-transmission time–space sequence diagram of the CRKP isolates in the HM department is presented in Figure 3.

Only one outbreak occurred at the end of Feb 2021, with four cases of CRKP HAIs, when the hematology ward was moved to the old tumor building, which had more beds and fewer medical staff (Figure 3). The source of the outbreak was case K22 (ST 11), and three cases (case K23 to case K25) were infected by CRKP. All four of them had CRKP BSIs, and case K22 and case K25 died. Case K22 shared a room with case K23, while case K24 shared a room with case K25. The implementation of various infection prevention strategies resulted in the prompt control of the outbreak within 2 weeks.

### Effects of the interventions

The study was conducted in three stages, with prevention and control interventions gradually upgraded at each stage (Figure 2). During the baseline period (Period 1), fewer CRKP isolates (0–6) were detected each year. Following an outbreak at the beginning of 2021, 14 CRKP cases were detected in that year. After Period 2, following the outbreak intervention bundles (March 2021), four patients brought CRKP into the HM department after the outbreak, but no cross-transmission occurred (Figure 3). Since Period 3, during the 2022 special CRKP control activity, a significant reduction in the overall rate of CRKP in the KP isolates was observed from 2021 to 2022 (46.7 and 12.9%, respectively).

Two cross-sectional active screenings of the rectal swabs were conducted in March and July 2021. A total of 107 rectal swabs for the CRKP screenings were collected, and only one new carrier (0.9%) was identified. The immediate surroundings of the patients with CRKP (cases K23, K24, K28, K36, and K38), including their bed unit and staff hands, were sampled five times from 2021 to 2022. Only one CRKP isolate (1.2%) was found on the floor of case K38’s room from all 85 samples (including bedside tables, bed stoppers, doorknobs, staff hands, and toilets). ATP bioluminescence testing of the surfaces was performed at the beginning of the outbreak, and 54.5% (12/22) of the tested items exceeded the threshold levels. Two weeks later, following enhanced environmental disinfection and terminal cleaning of all rooms, only 15.4% (4/26) of the tested items exceeded the threshold levels.

### The treatment and outcomes of CRKP

Of the 38 CRKP-isolated cases, seven were colonized and 31 had infections. The eradication rate of the colonized CRKP cases within 1 month after the first detection was 28.57% (2/7), while the eradication rate of the colonized CSKP cases was 42.31%. All seven colonized patients survived for more than 28 days. In the 31 patients with CRKP infections, the 28-day mortality rate was 38.7% (12/31). Of the 12 patients who died, 58.3% (7/12) died within 96 h after the first CRKP detection. Among the 12 deceased patients, nine died from septic shock caused by CRKP bacteremia and the other three died from multiple organ failure in the late stage of the tumor, with CRKP pulmonary infections simultaneously.

## Discussion

Our study reported that the total rate of CRKP in the KP isolates was 17.5% in the HM department over an 8-year period and described CRKP cross-transmission by integrating epidemiological data with WGS analysis. Our research was conducted in hospitals with high prevalence of CRKP, where the CRKP separation rate was greater than 60% in the ICUs (Li et al., 2022; Li et al., 2023). CRKP infections in China are relatively serious, as reported by Shaozhen Chen, who found that *K. pneumoniae* was the most common BSI isolates, with an infection rate as high as 4.7%. The rate of CRKP among the KP isolates was 17.5% in patients with HM receiving chemotherapy in Fujian Province, China (Chen et al., 2021). We found that the effective outbreak interventions, including single-room isolation, enhanced disinfection, and scrubbing CRKP-infected inpatients with chlorhexidine gluconate, played a pivotal role in controlling the outbreak and preventing the spread of HM-acquired CRKP infections. To the best of our knowledge, this is one of the first few comprehensive studies in China to include a bundle of interventions aimed at reducing the cross-transmission of HM-acquired CRKP infections.

In China, approximately 60% of CRKP strains produce KPC, with KPC-2 being the major variant of the KPC family (Hu et al., 2020). The widespread dissemination of KPC-2-producing CRKP has been found to be primarily limited to clone ST11 (Gu et al., 2019). Our research showed that the KPC-2-producing *K. pneumoniae* ST11 isolates were dominant (70.0%) in the HM department, emerging in 2014 and causing four cases of cross-transmission in the department. These findings were consistent with the results of the above-mentioned studies and the outcomes reported by our hospital’s microbiology team (Guo et al., 2022; Yan et al., 2019). The results showed that 79.1% (*n* = 170) of the CRKP isolates from 2012 to 2018 in the Respiratory Department carried the bla KPC-2 gene, and 89.4% of the isolates belonged to ST11 (Guo et al., 2022). The three strains of ST76, which were homologous, were distributed across three different wards, but they were detected within 3 months, indicating the possibility of cross-infection. However, in terms of the scale of CRKP transmission, HM departments typically experience one-to-one or one-to-two transmissions, unlike respiratory departments, which often experience large-scale CRKP infection outbreaks. We considered the following reasons: less invasive intubation procedures, fewer patients with CRKP infections being admitted to the HM department (Period 1), and enhanced intervention bundles (Periods 2 and 3).

Only 1.2% of the surroundings of the patients with CRKP infections were CRKP-positive in five environmental sampling sessions. The results of the environmental sampling sessions showed that cleaning and disinfection were well implemented and fewer inpatients with CRKP were infected, which was clearly better than in the ICU in our hospital. A total of 31.34% of CRKP bed units and 7.99% of environmental samples were CRKP-positive in our previous research on five major ICUs (Yan et al., 2019). The outbreak intervention bundles were effective, and we especially benefited from the single-room isolation of the patients with CRKP infections. Patients transferred from ICUs or other hospitals should be isolated in a single room, with enhanced disinfection and skin decolonization procedures. Since then, cases bringing CRKP into the HM department have not caused cross-transmission and no further outbreaks have occurred. Biehl et al. reported similar benefits of single-room isolation in a multicenter prospective study on HAIs (Biehl et al., 2019). The prevention of CRKP infections requires a multi-disciplinary approach involving hospital epidemiology, and the department should implement these measures effectively. Halaby et al. reported that in large-room ICUs, extended-spectrum β-lactamase-producing *K. pneumoniae* (ESBL-Kp) was continuously detected even after the intervention of contact isolation. However, the detection of ESBL-Kp decreased by 83.3% after the implementation of single-room isolation (Halaby et al., 2017).

CRKP active screening and control measures are effective in reducing the morbidity and mortality of CRKP infections in high-prevalence areas, such as Italy (Girmenia et al., 2015). In a nationwide study of Italian HSCT centers from 2010 to 2013, over 5,000 patients were screened for CRKP colonization prior to transplantation. Of these, 1% of autologous and 2.4% of allogeneic HSCT recipients were colonized with CRKP. Other studies in Italy reported that the CRKP colonization rate in patients with HM was 10.7% in 2012 and 52.5% in 2019 (Micozzi et al., 2017; Micozzi et al., 2021). However, our research showed that the detection rate of CRKP in the active screening was very low, at only 0.9% (1/107). A researcher from China, X L Huang, reported that the CRKP weekly screening colonization rate of patients with HM was 9.0% (36/401) in high CRKP prevalence areas of Zhejiang province from 2017 to 2019 (Huang et al., 2020). This difference may be related to varying patient colonization risks and different frequencies of screening between the two hospitals. Further studies are needed to validate these findings in China and assess the role of screening for CRKP in patients with HM or HSCT recipients.

Hematopoietic stem cell transplant (HSCT) recipients may be particularly vulnerable to CRKP infections because of chemotherapy-induced gastrointestinal mucositis, prolonged hospitalizations, neutropenia, and frequent use of broad-spectrum antibacterial agents (Satlin et al., 2014). Transplant centers in Italy reported that the post-transplant incidence of CRKP infections in allogeneic HSCT recipients increased from 0.4% in 2010 to 2.9% in 2013 (Girmenia et al., 2015). However, our research found that HSCT was a protective factor against CRKP, which may be related to the fact that the control group we studied consisted of patients with CSKP rather than uninfected individuals and that the HSCT transplant wards implemented stronger protective isolation measures. This conclusion needs to be validated with larger sample sizes in the future. The 28-day mortality rate among the patients with CRKP infections in our cohort (38.7%) was lower than that reported in other studies conducted among patients with HM in 13 Italian hematological wards (52.5%) (Trecarichi et al., 2016) and at a Chinese tertiary teaching hospital in Henan (80.6%) (Meng et al., 2022). In our study, more than half of the patients died within 96 h after the first CRKP detection, and the majority of them died from septic shock caused by CRKP bacteremia. This indicates that CRKP infections in patients with HM are very dangerous and require urgent medical care. Interestingly, among the 12 deceased patients, six strains were subjected to WGS, all of which were classified as ST11, KL47. In the study, three patients infected with hypermucoviscous ST76 strains (string test positive) survived. Although a long-term study across 29 centers from 17 cities in China reported that virulence genes in the plasmids of ST11-KL47 CRKP were evolving, driven by adaptive negative selection, enabling vertical chromosomal inheritance and conferring a survival advantage with low pathogenicity, our research findings are consistent with those of Gu D findings, showing that KL47-type strains can still cause high mortality (Wang et al., 2024; Gu et al., 2018).

Our study has a few limitations. Firstly, this was a single-center study, and the number of included patients was relatively small, so the results may not be generalizable to other institutions. Secondly, not all CRKP strains were whole genome sequenced, and genomic analysis was not performed on all strains. Thirdly, the interventions described in this study were bundled, making it difficult to determine the effectiveness of any single measure. Meanwhile, the implementation of the various interventions was not fully assessed during the study period.

## Conclusion

In summary, by integrating the epidemiological data with WGS analysis, we described CRKP cross-transmission in the HM department over an 8-year period. We found that the comprehensive outbreak interventions, such as single-room isolation, enhanced disinfection, and skin decolonization, played a pivotal role in controlling the spread of HM-acquired CRKP infections. KL47-type CRKP strains still exhibit high lethality.

## Data Availability

The original contributions presented in the study are publicly available. This data can be found here: https://www.ncbi.nlm.nih.gov/bioproject/PRJNA1195838; https://www.ncbi.nlm.nih.gov/bioproject/PRJNA1190865.

## Footnotes

^1^ https://mlstkv.insp.mx/

^2^ https://cge.cbs.dtu.dk/services/CSIPhylogeny/
